# Persistent Pain After Lithotripsy

**DOI:** 10.5811/westjem.2014.11.24467

**Published:** 2014-12-12

**Authors:** Ellen Jones, Kenneth DeKay

**Affiliations:** Carl R. Darnall Army Medical Center, Department of Emergency Medicine, Fort Hood, Texas

A 36-year old man presents to the emergency room five days after undergoing extracorporeal shock wave lithotripsy (ESWL) for a symptomatic 11mm left renal pelvis stone. The patient has persistent symptoms of severe left flank pain at presentation.

Plain abdominal radiography is obtained and shows a steinstrasse pattern of urolithiasis. In the 1980s, the German developers of ESWL observed that stone fragments could stack in a column formation in the ureter in a radiographic pattern that resembled a “stone street,” or steinstrasse in German (plural is *steinstrassen*) (Figure).^1^ This phenomenon is observed in 2–20% of ESWL patients and is a unique cause of ureteral obstruction that may present to the emergency department.^2^ Risk factors for the development of steinstrasse include large proximal stones, staghorn calculi, and pre-existing ureteral obstruction that may have caused permanent kinking of the ureter. Eighty-seven percent of steinstrassen occur in the distal ureter.

For asymptomatic cases of steinstrasse, conservative management is preferred with observation and serial imaging with plain radiography to follow progression of the stones and ultrasonography to evaluate for hydronephrosis. Sixty-five percent of steinstrassen will pass spontaneously over days to weeks.^2^ Tamsulosin has been more recently added as adjunctive therapy to improve outcomes in these asymptomatic cases. For symptomatic cases characterized by pain, fever, or hydronephrosis, more urgent urologic intervention is required through percutaneous nephrostomy or ureteroscopy with stent placement.

This particular patient underwent urgent ureteroscopy with stent placement the following day and had an uneventful recovery thereafter.

## Figures and Tables

**Figure:**
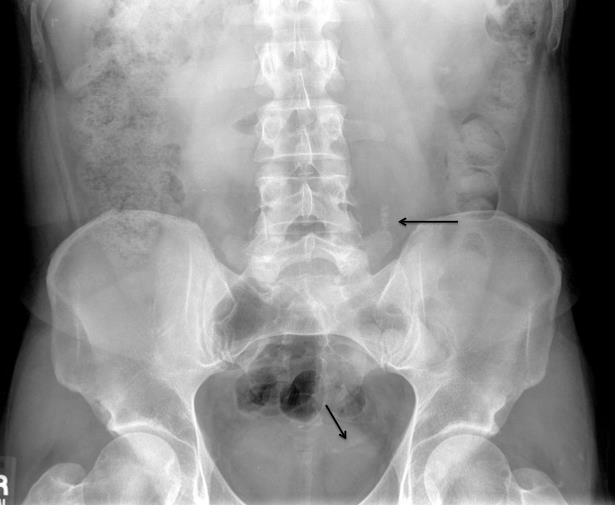
*Steinstrassen* seen in the left ureter.
